# Association Between Dyspeptic Symptoms and Eating Habits in the Colombian Population

**DOI:** 10.3390/nu18020308

**Published:** 2026-01-19

**Authors:** Julia María Alatorre-Cruz, Ricardo Carreño-López, Vanesa Vargas-Plaza, Lizbeth Barrios-Cortés, Yair Olovaldo Santiago-Sáenz, Claudia Fabiola Martínez-de la Peña, Norma Angélica Santiesteban-López, Graciela Catalina Alatorre-Cruz

**Affiliations:** 1 Instituto de Ciencias, Benemérita Universidad Autónoma de Puebla (BUAP), Puebla 72592, Mexico; julia_alatorre@yahoo.com.mx (J.M.A.-C.); claudia.martinez@correo.buap.mx (C.F.M.-d.l.P.); 2 Facultad de Medicina, Benemérita Universidad Autónoma de Puebla (BUAP), Puebla 72410, Mexico; lizbeth.barriosc@alumno.buap.mx; 3 Tecnología en Radiología e Imágenes Diagnósticas, Corporación Universitaria de Ciencias Empresariales, Educación y Salud (UNICORDSALUD), Montería 23001, Colombia; vargasplazavanesa@gmail.com; 4 Área Académica de Ingeniería Agroindustrial e Ingeniería en Alimentos, Instituto de Ciencias Agropecuarias, Universidad Autónoma del Estado de Hidalgo, Tulancingo 43775, Mexico; yair_santiago@uaeh.edu.mx; 5 Facultad de Administración, Benemérita Universidad Autónoma de Puebla (BUAP), Puebla 72592, Mexico; norma.santiesteban@correo.buap.mx; 6 Unidad de Investigación en Neurodesarrollo, Instituto de Neurobiología, Universidad Nacional Autónoma de México (UNAM), Querétaro 76230, Mexico

**Keywords:** dyspeptic symptoms, eating habits, Colombian population, predictors of symptoms, fast and fatty food, gastrointestinal inflammation, burning in the stomach

## Abstract

Background/Objectives: Functional dyspepsia (FD) is a gastrointestinal disorder typically treated by changes in diet and lifestyle. However, in the Colombian population, few studies have addressed its etiology and diagnosis. This exploratory study aimed to identify predictive variables associated with the presence of dyspeptic symptoms (DS). Methods: To address this, a self-survey was conducted evaluating sociodemographic characteristics, clinical history, and dietary habits. A DS index was calculated using participant’s clinical history to explore the characteristics of the groups with more and less DS (MDS and LDS groups). Additionally, a regression model was applied to identify the predictors of higher DS scores. Pooled data from the rolling, cross-sectional eating habits and DS survey between May and July of 2024. We enrolled 102 Colombian participants between 18 and 65 years old. Results: Significant differences were identified between MDS and LDS groups in occupation and dietary habits, with students exhibiting a higher DS index. Moreover, MDS exhibited greater consumption of fatty and fried foods than LDS groups. Regression analysis revealed that high intake of fatty foods and sesame were the best predictors of higher DS index. In contrast, the consumption of *Saccharomyces boulardii* probiotic and white onion was associated with better gastrointestinal health. Conclusions: Changes in dietary habits are associated with lower DS; the effect and its etiology might also depend on the participants’ occupation and nutritional habits.

## 1. Introduction

Functional dyspepsia (FD) is one of the most common multifactorial gastrointestinal disorders, affecting 8.4% of the global population, with a higher prevalence in women than in men [1,2,3,4,5]. FD is often underdiagnosed and undertreated [6]. The chronic or recurrent gastrointestinal symptoms of FD include pain, burning, bloating, early satiety, fullness, belching, nausea, or vomiting, without structural disease to explain them. These symptoms must occur at least three times per week for three to six months before diagnosis [3,4,7,8,9,10].

FD is currently divided into two subgroups based on the primary clinical pattern: postprandial distress syndrome (PDS) and epigastric pain syndrome (EPS). Some authors propose a third subgroup consisting of overlapping symptoms, referred to as PDS-EPS [3,4,7,8,9,11]. Consumption of certain foods typically triggers PDS and presents symptoms such as postprandial fullness, early satiety, loss of appetite, nausea, vomiting, and abdominal bloating. In contrast, EPS is characterized by epigastric pain or burning that occurs independently of food intake [2,7].

An FD diagnosis is established through a comprehensive medical history and physical examination during the critical period when the symptoms are present. Experts rely on the updated Rome IV criteria for functional gastrointestinal disorders to guide the diagnostic process, often supplemented with in situ tests. The gold standard for diagnosis is upper endoscopy with biopsy, which enables determination of the disease’s origin and severity [4,7,9]. Patients with FD commonly exhibit eosinophilic infiltration in the duodenum, particularly in cases of postprandial distress syndrome (PDS). Additionally, FD has been associated with circulating T cells in the small intestine, elevated cytokine levels, and elevated tumor necrosis factor-α (TNF-α) levels, suggesting underlying intestinal inflammation [12]. Other key contributors to FD pathophysiology include abnormal central modulation, visceral hypersensitivity, and increased intestinal mucosal permeability, often linked to disruptions in the gut brain axis [7].

FD treatment typically involves dietary and lifestyle modifications, pharmacological therapy such as proton pump inhibitors (PPIs), and, in some cases, psychological treatment depending on the underlying cause [2,7,8]. Treatment remains challenging due to the unclear etiology of dyspepsia. However, certain factors have been associated with the condition, including chronic use of nonsteroidal anti-inflammatory drugs (NSAIDs), consumption of highly spiced foods, *Helicobacter pylori* infection acquired through contaminated food, lack of access to clean drinking water, and other environmental factors [4,7,8,10,11,13,14].

Previous studies have demonstrated a significant impact of dietary habits on FD pathophysiology, triggering or exacerbating symptoms such as postprandial fullness, early satiety, epigastric pain, acid and duodenal hypersensitivity, food allergies, and intolerance or sensitivity to certain foods [5,7,11]. These symptoms have been associated with increased caloric intake and inadequate eating behaviors, such as late-night consumption of processed snacks, consistently skipping one or more meals, frequent intake of large portions, high liquid intake while fasting, and rapid eating without proper chewing [5,15]. An imbalance in macronutrient consumption, such as high-fat, protein, or simple carbohydrate diets, has been associated with the exacerbation of dyspeptic symptoms (DS). Moreover, specific foods have been involved in DS, including spicy foods, industrialized juices, carbonated beverages, wheat products, gluten, legumes, watermelon, red peppers, caffeinated products, tea, alcohol, chocolate, processed meats, vinegar-based pickles, salty food, macaroni, fast food, and acidic foods [7,11,15,16]. On the other hand, certain foods and supplements have been recognized to alleviate DS, including acid suppressants, prokinetics, and neuromodulators, as well as fruits such as apples, rice, yogurt, honey, rock sugar, bread, caraway seeds, quince, and nuts [16]. Other effective options include ginger [17], medicinal herbs [18,19], nutraceutical supplements containing perilla and ginger extracts [20], supplements with ginger and artichoke extracts [21], and mastic gum, among others [7,11,16,22].

Recent studies indicate that a significant proportion of the Colombian population experiences DS, particularly in urban areas. According to the Ipsos Global Health Services Monitor, 84% of Colombians reported digestive discomfort related to imbalances in the gut microbiota, which contribute to DS. The prevalence of this condition varies across cities in the country, with Barranquilla and Medellín reporting the highest rates, followed by Bogotá and Cali. Despite its high prevalence, FD in Colombia remains underdiagnosed. Many patients do not seek medical attention due to the normalization of digestive symptoms or limited access to specialized healthcare.

Furthermore, appropriate treatment is not always available, and empirical treatments often target symptoms without considering the underlying cause of DS. The gap in healthcare access between urban and rural areas exacerbates the situation. People living in rural or underserved regions face significant barriers to obtaining proper diagnosis and treatment, worsening the dyspepsia situation.

Therefore, this study had two primary aims: the first was to characterize differences between participants with more and less DS in sociodemographic, environmental, and dietary factors, and gastrointestinal and non-communicable diseases (such as diabetes and hypertension). The second, equally important, was to explore predictor variables related to eating habits that increase the risk of DS in the Colombian population.

## 2. Materials and Methods

### 2.1. Participants

The present study enrolled 102 Colombian participants aged 18 to 65 years. All of them are native Spanish speakers with at least 9 years of scholarship and internet access. In this study, health condition was assessed through an online survey; therefore, volunteers were not examined by a physician. The participants who stated they suffered from chronic diseases such as cancer or *Helicobacter pylori* infection were excluded from this study. The sample was randomly selected in different regions in Colombia (see Appendix A). The study was conducted in accordance with the Declaration of Helsinki, and participants were informed of their rights and provided written informed consent to participate. The results are part of the protocol approved by the BUAP, registration number 042.

### 2.2. Procedure

The data were obtained from a self-administered survey directed to the Colombian population. This entailed 55 items distributed in four sections: (1) identification data; (2) anthropometric data; (3) medical records; and (4) food intake frequency in the last month, including fruits, vegetables, cereals, legumes, spices, and beverages (See Appendix B). The present study employed an exploratory, cross-sectional design and used non-probabilistic convenience sampling. A pilot group of 40 subjects participated in the survey; all participants reported a thorough understanding of the items. They also reported being comfortable with the process and the completion time. The pilot group responded to 100% of the questions in around 25 min. We performed a statistical analysis to validate the survey using a chi-square test. The factor analysis technique used an orthogonal rotation, “Varimax”. We also measured the internal consistency of each item within each factor using Cronbach’s alpha (0.857). We also computed the Kaiser–Meyer–Olkin (KMO) statistics (0.748) and Bartlett’s sphericity test (X^2^ (21) 468.295, *p* = 0.0001). A DS index was generated to assess symptom severity based on participants’ responses to our survey (see Table 1).

Additionally, the DS index positively correlated with the ROMA IV criteria (Bristol scale, bowel moving frequency) (*p* = 0.04), which might support preliminary convergent validity of our DS index.

### 2.3. Data Analysis Methods

#### 2.3.1. Cluster Analysis

To explore this study’s first aim. A K-means clustering was performed to separate our sample into two groups. The clustering analyses resulted in 69 participants (Mean (M) = 30.0, ±2.7; range: 23–38) with a higher DS score (MDS) and 33 participants (M = 13.3, ±3.8; range: 4–21) with a lower DS score (LDS). After bootstrap analysis conducted with a sample size of 1000, significant differences in DS scores remain between the MDS and LDS groups (t(100,47) = 24.3, *p* = 0.000).

#### 2.3.2. Comparisons Between Less vs. More DS (LDS vs. MDS)

Some conditions, such as sex, civil status, scholarship, occupation, and frequency of food consumption, were compared between DS groups using aχ^2^ test (i.e., vegetables, fruits, red meat, white meat, eggs, dairy products, soft drinks, pasta, and fast food). We also compared the frequency of consumption of fatty foods (i.e., arepas, fried foods, fritanga, buñuelos, cakes) between groups using aχ^2^ test. The age was compared between groups with the Mann–Whitney U test (Kolmogorov–Smirnov test, *p* = 0.001), and body mass index (BMI) with the *t*-test (Kolmogorov–Smirnov test, *p* = 0.2). The Kendall Tau-C model was also calculated to explore the relationship between DS groups (i.e., LDS and MDS) and BMI category. *p*-values resulting from a set of comparisons were corrected by the FDR method. We report results surviving FDR correction (*p*-values < 0.05).

#### 2.3.3. Exploratory Predictors of the DS Index

Linear regression analysis was performed to identify the relationship between the DS index and demographic, anthropometric data (BMI), comorbidities, and the frequency of food consumption. The DS index was included as the dependent variable. Sex, age, and scholarship, diseases (i.e., diabetes, hypertension, hypercholesterolemia, hypertriglyceridemia, nonspecific ulcerative colitis (UC), irritable bowel syndrome, and obesity, drug addiction), frequency of consumption of food (i.e., fruits, vegetables, red and white meats, spices, cereals, legumes, fermented foods and commercial products containing probiotics) were included as independent variables. The Kolmogorov–Smirnov test was used to assess the normality of the variables. Descriptive analyses were performed, and the results are presented as means and standard deviations (SD), as well as proportions and/or percentages, depending on the type of variable analyzed. The (χ^2^) test was used to compare categorical variables, e.g., BMI vs. DS Index (dichotomous). A regression analysis was conducted to identify which variables positively or negatively predict the outcome variable of interest (DS). We performed a multicollinearity analysis before including predictor variables in the regression model, following the next tests: correlation analyses, tolerance values, variance inflation factors, and condition indices. Variables with values outside the parameter range were excluded from the regression analysis (see Supplementary Materials).

## 3. Results

### 3.1. Participant Characteristics

Table 2 illustrates demographic, anthropometric, and distribution of non-communicable diseases for LDS and MDS groups.

### 3.2. Demographic and Anthropometric Comparisons Between LDS and MDS Groups

Analyzing our results, shown that the DS groups did not differ in sex (χ^2^ (1) 0.4, *p* = 0.5), civil status (χ^2^ (3) 7.5, *p* = 0.06), scholarship (χ^2^ (4) 6.0, *p* = 0.2), and BMI (χ^2^ (3) 3.7, *p* = 0.3; Kendall Tau C = 0.04, *p* = 0.7). However, they differed in occupation (χ^2^ (7) 14.2, *p* = 0.04; V of Cramer = 0.4, *p* = 0.04). We also found a higher number of students in the MDS than in the LDS group. Additionally, the groups did not differ in age (U (*p* = 0.1).

### 3.3. Frequency of Food Consumption of the LDS and MDS Groups

When analyzing the food consumption of LDS and MDS group we found that the DS groups did not differ in the consumption of vegetables (χ^2^ (7) 9.4, *p* = 0.22), fruit (χ^2^ (7) 13.0, *p* = 0.07), white meat (χ^2^ (7) 6.3, *p* = 0.5), egg (χ^2^ (7) 10.9, *p* = 0.1), dairy products (χ^2^ (7) 8.6, *p* = 0.3), soft drinks (χ^2^ (7) 2.8, *p* = 0.9), and pasta (χ^2^ (7) 3.0, *p* = 0.09). However, they differed in their consumption of red meat (X^2^ (7) = 10.2, *p* = 0.02) and fast food (χ^2^ (4) = 33.8, *p* = 0.0001), and fatty foods: arepas (χ^2^ (4) 21.8, *p* = 0.001), fried food (χ^2^ (4) 33.85, *p* = 0.001), fritanga (χ^2^ (4) 11.5, *p* = 0.02), buñuelos (χ^2^ (4) 11.7, *p* = 0.02), cakes (χ^2^ (4) 14.5, *p* = 0.006), with a higher frequency of consumption in the MDS group than in the LDS group. Table 3 shows the frequency of consumption for all food groups.

### 3.4. Exploratory Predictors of the DS

The regression model (sum of squares = 2892.3; root mean square = 723.0; Effect size = 0.15; Standardized residual (Kolmogorov–Smirnov test, *p* = 0.2; homoscedastic) indicated that the consumption of some foods might be associated with the DS index. The supplements, such as *Saccharomyces boulardii* and white onion, were associated with a lower DS index. In comparison, the consumption of fatty foods and sesame may be linked to a higher DS index. These results matched previous analyses of food consumption frequency (See Table 4).

## 4. Discussion

This study aimed to develop an exploratory instrument to link DS to medical records, sociodemographic variables, and dietary habits. Moreover, as expected, the frequency of food consumption was associated with the DS. However, we did not find medical records or sociodemographic variables as predictors. Therefore, our findings require an additional explanation.

### 4.1. Demographic and Anthropometric Data

As we expected, there were occupation differences between the LDS and MDS groups, as reported in previous studies [23,24]. The sample consisted of a higher number of students belonging to the MDS group, which matched prior studies reporting a positive correlation between higher education level and a higher prevalence of gastric disorders in Asian adults [23,24]. We suggest that our results may be explained by the fact that most students have a high level of education, which leads them to perceive their gastric condition differently. Moreover, students and workers with high responsibilities often have unhealthy lifestyles and dietary habits, which can lead to gastrointestinal disorders. Moreover, the occupational stress found in subjects with high educational levels could affect these disorders [25], which is also evident in our students, who belong to the MDS group.

On the other hand, contrary to Zhang et al.’s (2017) [24] findings, we did not find an association between DS and married status. From a socioeconomic perspective, married status could increase stress, responsibilities, and unhealthy dietary habits and increase the probability of suffering from DS. In the present study, most participants were single, which hampers our ability to examine any relationship between marital status and DS.

Differences in anthropometric data between the LDS and MDS groups were anticipated, as a previous study reported a strong association between underweight status and acute DS [26,27]. However, this association was not observed. The BMI distribution in the present sample may account for this finding, as most participants were of normal weight, and only seven were classified as underweight, which was insufficient to establish the previously reported relationship.

### 4.2. Frequency of Food Consumption and DS

Our results showed that food intake was associated with DS; specifically, the MDS group had higher intake of red meat, fast food, and fatty foods than the LDS group. These findings align with previous studies reporting associations between a higher DS index and higher red meat consumption [16,26], fast food [15], and fatty foods [3,5,7,10,15,26,27,28]. We suggest, as other studies have, that this type of food may negatively impact specific biological mechanisms involved in appetite regulation and digestion [7,26].

### 4.3. Exploratory Predictors of the DS

As expected, our results showed that high intake of fatty foods and sesame might predict higher DS, aligning with previous studies [3,5,7,10,15,26,27,29,30,31]. Fatty food seems to exacerbate symptoms such as fullness, bloating, nausea, and discomfort, enhancing sensitivity to gastric distension [3,5,10,14,27] and abnormalities in motility [27]. Prior studies have described at least two different mechanisms that might explain the association between fatty food intake and more severe DS found in this study: (1) high levels of cholecystokinin (CCK) and (2) the formation of acrolein. In the first mechanism, elevated circulating CCK levels have been associated with feelings of fullness or satiety [7]. Interestingly, CCK plays a crucial role in the symptoms of DS observed after consuming a meal rich in fat or protein. The CCK receptor antagonist dexloxiglumide seems to alleviate lipid-induced symptoms following intraduodenal lipid infusion in patients with DS [32]. In the second mechanism, when foods are fried at high temperatures, the cooking oil undergoes oxidation, resulting in the formation of acrolein. This pungent compound irritates the oral and nasal mucosa and contributes to the bitter taste of degraded oil, which may, in turn, contribute to the onset of postprandial fullness [5].

On the other hand, the adverse effects of sesame on DS may depend on the preparation method [31]. High-temperature processing of sesame can alter its phenolic compounds or the oxidation state of its fatty acids, potentially irritating the gastric mucosa in susceptible individuals [31] and activating their pro-inflammatory and pro-oxidant mechanisms [33].

Another interesting result was that a higher intake of *Saccharomyces boulardii* was associated with a lower DS index, which also warrants additional explanation. *Saccharomyces boulardii’s* effect on the DS index may be supported by its probiotic qualities, which have been widely recognized as beneficial for gastrointestinal health [34,35,36]. This supplement seems to modulate host immune responses, stabilizing intestinal epithelial barrier integrity, inhibiting pathogen binding, and promoting the secretion of digestive enzymes, thereby enhancing nutrient absorption and reducing inflammation [34,37].

The high intake of white onions was associated with a lower DS score. This fact may be explained by bioactive compounds such as quercetin, organosulfur compounds, and fructans, which have anti-inflammatory, antioxidant, and antimicrobial properties against pathogens such as *H. pylori* [38,39].

## 5. Conclusions

Changes in dietary habits can alleviate DS; however, because dietary habits differ across regions, their etiology will depend on participants’ age and occupations. Moreover, for this Colombian population, high intake of fatty foods and acidic foods might trigger a higher DS index. In contrast, consuming *Saccharomyces boulardii* probiotics will benefit gastrointestinal health.

## 6. Limitations

The present study has several inherent limitations. First, its cross-sectional design precludes the establishment of causal relationships or definitive conclusions regarding predictors of the DS index. Second, participants were not clinically evaluated by a physician; therefore, the presence of undiagnosed or comorbid medical conditions cannot be ruled out. In addition, the sample was recruited by convenience and was predominantly composed of students, which limits the external validity and generalizability of the findings. Furthermore, we did not assess 24 h dietary intake, eating behaviors (e.g., chewing speed, portion sizes, or meal skipping), levels of physical activity, fatigue, psychological factors (such as anxiety and stress), or sleep disorders. Consequently, the findings and interpretations of this study should be considered with caution.

## Figures and Tables

**Table 1:** DS index calculation.

**Item**	**Raw Core**	DS Index formula DS Index = Raw scores [(1 × 2) + 3 + 4 + (6 × 6.1) + 6.2 + 7 + 8]	**Weight of Factorial Analysis**
1. Do you suffer from gastritis or dyspepsia? * 2. Who diagnosed you with gastritis or dyspepsia?	Presence = 1 Absence = 0 ND = 0 Myself = 1 Physician = 2		0.845 × 0.848
3. Do you have heartburn?	Presence = 1 Absence = 0		0.757
4. Do you associate stomach burning with any specific factor?	Stress = 1 Unhealthy food intake habits = 2 ND = 0		0.648
5. Do you have a burning in the stomach?	Presence = 1 Absence = 0		0.662
6. Do you avoid any food? *	Presence = 1 Absence = 0		0.800 × 0.900
6.1 Which foods do you avoid?	ND = 0 Make me feel unhealthy = 3 It is prohibited for me = 2 I don’t like it = 1		
6.2 Indicate which foods you avoid	0–27		
7. Do you have gastrointestinal inflammation after meals?	Presence = 1 Absence = 0		0.688
8. Do you have nausea after eating?	Presence = 1 Absence = 0		0.617

Note: DS = Dyspeptic symptoms, ND = No data. The symbol * means multiplication of Weight of Factorial Analysis of questions (1 × 2) and (6 × 6.1).

**Table 2:** Demographic, anthropometric, and non-communicable disease data for the LDS and MDS groups.

	LDS		MDS	
	**Mean (SD)**		**Mean (SD)**	
**Age (years old)**	28.3 (11.0)		23.6 (6.4)	
	***n***	**(%)**	***n***	**(%)**
**Sex**	15 Female 18 Male	(45.5) (54.5)	36 Female 33 Male	(52.2) (47.8)
**Scholarship**				
High school	6	(18.2)	20	(29.0)
Technical	2	(6.1)	12	(17.4)
Technologist	2	(6.1)	1	(1.4)
Undergraduate	22	(66.7)	35	(50.7)
Postgraduate	1	(3.0)	1	(1.4)
**Civil status**				
Single	17	(51.5)	43	(62.3)
Married	7	(21.2)	3	(4.3)
Free union	9	(27.3)	22	(31.9)
Divorced	-	-	1	(1.4)
**Occupation**				
Professional	14	(42.4)	11	(15.9)
Housewife	-	-	4	(5.8)
Employee	2	(6.1)	5	(7.2)
Self-Employed	1	(3.0)	8	(11.6)
Businessman	-	-	1	(1.4)
Student	11	(33.3)	37	(53.6)
Military	-	-	2	(2.9)
Policeman	1	(3.0)	1	(1.4)
**Diseases**				
Diabetes mellitus	3	(9.1)	1	(1.4)
Hypertension	3	(9.1)	1	(1.4)
Hypercholesterolemia	5	(15.2)	7	(10.1)
Hypertriglyceridemia	3	(9.1)	3	(4.3)
IBS (irritable bowel syndrome)	-	-	3	(4.3)
**BMI**				
Underweight	4	(12.1)	3	(4.3)
Normal Weight	14	(42.4)	41	(59.4)
Overweight	12	(36.4)	21	(30.4)
Obesity	3	(9.1)	4	(5.8)

Note: LDS = Less dyspeptic symptoms score, MDS = More dyspeptic symptoms score, SD = Standard deviation, BMI = Body mass index. “-“ No data.

**Table 3:** Distribution of food groups in LDS and MDS.

	LDS_Never (%)	LDS_1–3 t Week (%)	LDS_ > 4 t Week (%)	MDS_Never (%)	MDS_1–3 t Week (%)	MDS_ > 4 t Week (%)
Vegetables	0	24.2	75.7	8.7	39.1	52.1
Fruit	0	51.5	48.5	4.3	65.2	23.1
Red meat *	6.1	81.8	12.1	0	69.6	30.4
White meats	0	69.8	30.4	1.4	81.8	17.3
Egg	0	63.6	36.3	4.3	43.4	52.1
Dairy products	6.1	60.6	33.3	5.8	71	23.1
Soft drinks	3	48.5	48.5	1.4	37.6	60.9
Pasta	6.1	84.8	9.1	1.4	82.6	15.8
Fast food ***	42.4	36.4	21.2	4.3	82.6	42
Arepas ***	0	33.4	9.1	0	62.3	17.4
Fried foods ***	0	24.3	12.1	0	68.1	17.4
Fritanga *	0	27.3	3.0	0	39.1	8.7
Buñuelos *	0	30.4	6.1	0	37.7	11.6
Cakes ***	0	21.2	6.1	0	27.5	13

Note: LDS = Less dyspeptic symptoms score, MDS = More dyspeptic symptoms score, t = times per week. * *p*-values < 0.05; *** *p*-values < 0.001.

**Table 4:** Food consumption predictors of the DS index.

Exploratory Predictors of DS Index	Standardized Coefficients	t	*p*-Value	F	R^2^	*p*	VIF
	Beta						
(Constant)	4.6	0.9	0.3	16.1	0.4	0.001	
How many times per week do you consume fatty food?	0.5	6.5	0.000				1.1
*Saccharomyces boulardii* probiotic	−0.3	−4.2	0.000				1.1
White onion	−0.3	−3.7	0.000				1.0
Sesame	0.2	3.1	0.002				1.1

Note: DS Index = Dyspeptic symptoms index.

**Figure A1:**
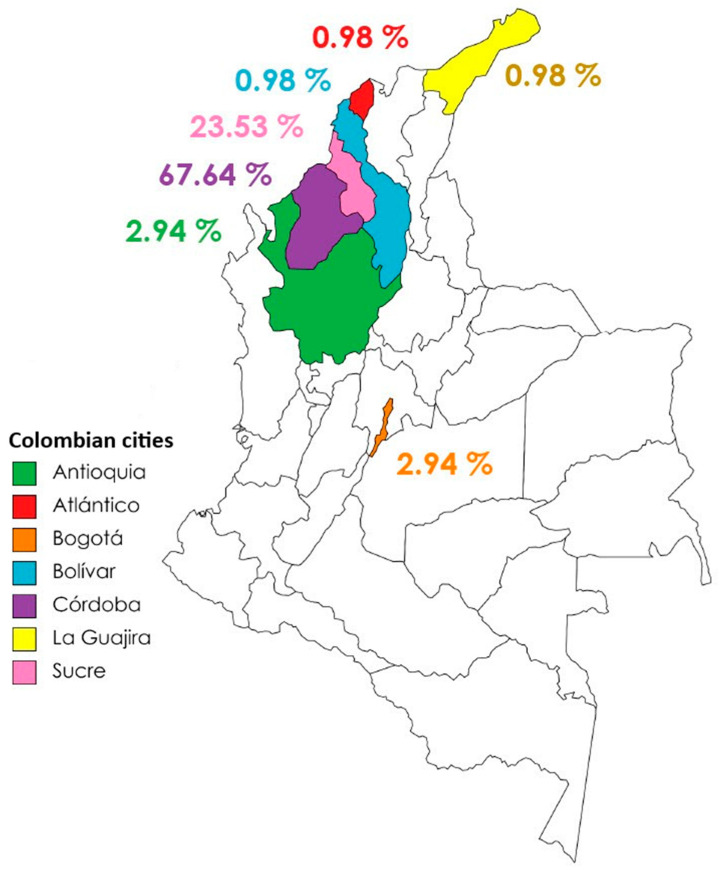
Regional distribution of the study in the Colombian population.

## Data Availability

The data are available at the following link: https://figshare.com/s/c493b54f673aaba71b27 (accessed on 14 January 2026).

## Supplementary Materials

The following supporting information can be downloaded at: https://www.mdpi.com/article/10.3390/nu18020308/s1.

## Abbreviations

The following abbreviations are used in this manuscript:

FD	Functional dyspepsia
DS	Dyspeptic symptoms
PDS	Postprandial distress syndrome
EPS	Epigastric pain syndrome
TNF	Tumor necrosis factor
PPIs	Proton pump inhibitors
NSAIDS	Nonsteroidal anti-inflammatory drugs
LDS	Less dyspeptic symptoms
MDS	More dyspeptic symptoms
UC	Ulcerative colitis

## Appendix B

**Table A1:** Self-administered survey directed to the Colombian Population.

Demographic and Health Information	Información de Salud y Demográficos
Personal Information	Información personal
Sex	Sexo
Age	Edad
Marital status	Estado civil
Nationality	Nacionalidad
Place of residence	Lugar de residencia
Anthropometric Data	Datos antropométricos
Height (m)	Estatura (m)
Weight (kg)	Peso (kg)
Socioeconomic and Occupational Data	Datos ocupacionales y socioeconómicos
Occupation	Ocupación
Education level	Nivel de educación
Metabolic and Cardiovascular Conditions	Condiciones cardiovasculares y metabólicos
Do you have type 2 diabetes mellitus?	¿Usted padece diabetes mellitus tipo 2?
Do you have high cholesterol levels?	¿Usted padece niveles altos de colesterol?
Do you have high triglyceride levels?	¿Usted padece niveles de triglicéridos altos?
Do you have hypertension (high blood pressure)?	¿Usted padece hipertensión?
Gastrointestinal Disorders	Desordenes gastrointestinales
Do you have gastritis?	¿Usted padece de gastritis?
Who diagnosed your gastritis?	¿Quién le diagnosticó la gastritis?
Do you experience acid reflux (heartburn)?	¿Ha experimentado reflujo?
Do you experience stomach burning?	¿Ha experimentado acidez estomacal?
Do you associate stomach burning with any specific factor?	¿Ha asociado la acidez estomacal con algún factor específico?
Do you have irritable bowel syndrome (IBS)?	¿Usted padece síndrome de intestino irritable?
Do you have nonspecific ulcerative colitis?	¿Usted padece colitis ulcerativa inespecífica?
Do you suffer from constipation?	¿Usted padece estreñimiento?
How many times do you have a bowel movement per day?	¿Cuántas veces evacua, hace del baño heces por día?
Do you experience bloating after eating?	¿Experimenta ardor de estómago después de comer?
Do you experience nausea after eating?	¿Experimenta náuseas después de comer?
Other Chronic Diseases	Otras enfermedades crónicas
Do you have any type of cancer? (If yes, specify type)	¿Usted padece algún tipo de cáncer? Si es así, especifique el tipo.
Do you have any other medical conditions?	¿Usted padece alguna otra condición médica?
If yes, which ones?	Si es así, cuál?
Dietary Habits	Hábitos dietarios
Liters of water per day	¿Cuántos litros de agua usted bebe al día?
Do you drink coffee? (Yes/No)	¿Usted toma café? Si/No
How many times per week?	¿Cuántas veces por semana bebe café?
How many cups per day?	¿Cuántas tazas de café toma al día?
Do you drink alcohol? (Yes/No)	¿Usted consume bebidas alcohólicas?
Type and quantity of alcoholic beverages consumed	¿Qué tan frecuente consume bebidas alcohólicas y que cantidad?
Consumption of Frequency Food Groups	Frecuencia de consumo de grupos alimentarios
How many times per week do you consume:	¿Cuántas veces por semana consume?
Vegetables	Verduras
Fruits	Frutas
Red meat	Carnes rojas
White meat (poultry, fish, etc.)	Carnes blancas (pollo, pescado)
Eggs	Huevo
Dairy products	Productos lácteos
Specific Food Frequency	Frecuencia de consumo específicos
How frequently do you consume the following foods?	¿Qué tan frecuentemente consume los siguientes alimentos?
Fruits (e.g., grapes, blueberries, peaches, raspberries, grapefruit, kiwi, oranges, etc.)	Frutas (uvas, duraznos, blueberries, zarzamora, frambuesa, kiwi, fresa, naranjas, etc.)
Vegetables (e.g., spinach, carrots, bell peppers, mushrooms, tomatoes, onions, etc.)	Vegetales (espinacas, zanahorias, champiñones, pimientos, tomate, jitomate, cebolla, etc.)
Grains and legumes (e.g., rice, oats, barley, wheat, corn, lentils, beans, etc.)	Granos y legumbres (Arroz, avena, trigo, maíz, lentejas, frijoles etc.)
Nuts and seeds (e.g., almonds, walnuts, peanuts, chia, flaxseeds, etc.)	Nueces y semillas (Almendras, nueces, chía, almendras, linaza etc.)
Spices and Seasonings Consumption	Consumo de especias y sazonadores
How frequently do you consume these spices? (e.g., parsley, cilantro, oregano, garlic, paprika, cumin, cinnamon, etc.)	¿Qué tan frecuente usted consume las siguientes especias? cilantro, perejil, orégano, ajo, paprika, cúrcuma, canela
Do you avoid any spices? If yes, why?	¿Usted evita alguna especia? ¿Si es así, por qué?
Fermented Foods Consumption	Consumo de alimentos fermentados
How frequently do you consume fermented foods? (e.g., pickled olives, blue cheese, parmesan, yogurt, natilla, etc.)	¿Qué tan frecuente, usted consume alimentos fermentados? (ej. Aceitunas, queso azul, parmesano, yogurt, natilla, etc.)
Do you avoid any fermented foods? If yes, why?	¿Usted evita algún alimento fermentado? ¿Si es así, por qué?
Probiotics and Supplements	Suplementos y probióticos
Do you consume probiotic supplements?	¿Usted consume algún suplemento con probióticos?
If yes, which type and for how long?	Si es así, ¿qué tipo y por cuánto tiempo lo consume?
Beverages Consumption	Consumo de bebidas
How frequently do you consume these beverages?	¿Qué tan frecuente, usted consume las siguientes bebidas?
Tea varieties (e.g., green tea, black tea, Chamomilla, mint tea, etc.)	Variedades de té: negro, verde, Chamomilla, menta, etc.
Juices (e.g., orange, mango, apple, cherry, etc.)	Jugos: naranja, mango, manzana, cereza, etc.
Other beverages (e.g., agua miel, cinnamon-infused drinks, etc.)	Otras bebidas: agua miel, infusiones de canela, etc.
Do you avoid any beverages? If yes, why?	¿Usted evita alguna bebida? ¿Si es así, por qué?
Consumption of Processed and Fast Foods	Consumo de comidas fritas y procesadas
How many times per week do you consume:	¿Cuántas veces por semana usted consume los siguientes alimentos?
Soft drinks	Bebidas carbonatadas, refrescos
Pasta	Pastas
Fast food	Comida rápida
Fatty foods (e.g., buñuelos, pasteles, arepas, etc.)	Preparaciones fritas: buñuelos, pasteles, arepas, etc.
