# Supplementary material for: Association Between Dyspeptic Symptoms and Eating Habits in the Colombian Population
Source: Nutrients. 2026 Jan 19;18(2):308. doi: 10.3390/nu18020308 (PMC12844606; doi:10.3390/nu18020308)
Supplement: Supplementary file 1 [file nutrients-18-00308-s001.zip › nutrients-4035080-supplementary.pdf]

Excluded variables							
	In beta	t	sig	Partial correlation	Collinearity statistics		
					Tolerance	VIF	Minimum tolerance
Sex	.011c	0.124	0.901	0.013	0.987	1.013	0.912
Age	-.044c	-0.481	0.632	-0.049	0.869	1.151	0.804
Weight (kg)	-.044c	-0.516	0.607	-0.052	0.99	1.011	0.916
BMI	-.031c	-0.36	0.72	-0.036	0.996	1.004	0.921
Do you have overweight or obesity?	.212c	2.481	0.015	0.243	0.949	1.054	0.893
Do you have type 2 diabetes mellitus? (High blood sugar)	-.057c	-0.662	0.509	-0.067	0.995	1.005	0.92
Do you have hypertension? (High blood pressure)	-.054c	-0.625	0.533	-0.063	0.97	1.031	0.896
Do you have high cholesterol?	.021c	0.245	0.807	0.025	0.989	1.011	0.914
Do you have dyslipidemia (high triglycerides)?	-.003c	-0.031	0.975	-0.003	0.979	1.022	0.906
How often do you eat these foods? [Grapes]	-.172c	-2.017	0.046	-0.2	0.969	1.032	0.903
How often do you eat these foods? [Plums]	.189c	2.038	0.044	0.202	0.822	1.216	0.82
How often do you eat these foods? [Blueberries]	-.009c	-0.097	0.923	-0.01	0.912	1.097	0.843
How often do you eat these foods? [Peaches]	-.107c	-1.229	0.222	-0.123	0.963	1.039	0.89
How often do you eat these foods? [Raspberries]	.008c	0.084	0.933	0.009	0.928	1.078	0.861
How often do you eat these foods? [Blueberries]	-.017c	-0.185	0.853	-0.019	0.873	1.146	0.806
How often do you eat these foods? [Grapefruit]	.027c	0.299	0.766	0.03	0.931	1.074	0.86
How often do you eat these foods? [Kiwi]	.001c	0.016	0.987	0.002	0.896	1.116	0.846
How often do you eat these foods? [Oranges]	.040c	0.449	0.655	0.045	0.949	1.054	0.884
How often do you eat these foods? [Guava]	.066c	0.718	0.475	0.072	0.857	1.167	0.847
How often do you eat these foods? [Strawberries]	-.009c	-0.1	0.92	-0.01	0.933	1.072	0.863
How often do you eat these foods? [Pomegranates]	-.026c	-0.302	0.763	-0.03	0.959	1.043	0.889
How often do you eat these foods? [Cherry]	.022c	0.248	0.805	0.025	0.97	1.03	0.902
How often do you eat these foods? [Mandarin Oranges]	.014c	0.15	0.881	0.015	0.898	1.114	0.842
How often do you eat these foods? [Yellow Bananas]	-.079c	-0.906	0.367	-0.091	0.974	1.026	0.913
How often do you eat these foods? [Limes]	.046c	0.5	0.618	0.05	0.864	1.157	0.808
How often do you eat these foods? [Mamey]	.005c	0.06	0.952	0.006	0.914	1.094	0.846
How often do you eat these foods? [Soursop]	-.021c	-0.237	0.813	-0.024	0.9	1.111	0.834
How often do you eat these foods? [Papaya]	-.049c	-0.547	0.586	-0.055	0.909	1.1	0.85
How often do you eat these foods? [Dragon Fruit]	.022c	0.243	0.808	0.025	0.912	1.097	0.843
How often do you eat these foods? [Mushrooms]	-.056c	-0.621	0.536	-0.063	0.9	1.111	0.834
How often do you eat these foods? [Peppers]	.092c	1.058	0.293	0.106	0.954	1.048	0.893

How often do you eat these foods? [Carrot]	-.077c	-0.889	0.376	-0.089	0.978	1.023	0.903
How often do you eat these foods? [Chili pepper]	-.001c	-0.016	0.987	-0.002	0.98	1.021	0.913
How often do you eat these foods? [Lettuce]	-.085c	-0.968	0.335	-0.097	0.949	1.053	0.9
How often do you eat these foods? [Radish]	.006c	0.066	0.947	0.007	0.896	1.117	0.828
How often do you eat these foods? [Celery]	.027c	0.307	0.759	0.031	0.961	1.04	0.888
How often do you eat these foods? [Green tomatoes]	-.047c	-0.543	0.589	-0.055	0.995	1.005	0.92
How often do you eat these foods? [Prickly pear]	.014c	0.141	0.888	0.014	0.7	1.429	0.658
How often do you eat these foods? [Sapote]	-.072c	-0.833	0.407	-0.084	0.974	1.027	0.907
How often do you eat these foods? [White onion]	-.248c	-2.981	0.004	-0.288	0.976	1.025	0.905
How often do you eat these foods? [Red onion]	-.140c	-1.629	0.106	-0.162	0.976	1.025	0.908
How often do you eat these foods? [Spring onions]	-.202c	-2.41	0.018	-0.237	0.991	1.009	0.916
How often do you eat these foods? [Broccoli]	-.034c	-0.387	0.7	-0.039	0.929	1.077	0.869
How often do you eat these foods? [Green beans]	-.062c	-0.709	0.48	-0.071	0.959	1.043	0.888
How often do you eat these foods? [Pumpkin]	.004c	0.046	0.963	0.005	0.859	1.164	0.8
How often do you eat these foods? [Spinach]	.109c	1.252	0.214	0.125	0.952	1.051	0.88
How often do you eat these foods? [Cauliflower]	-.025c	-0.271	0.787	-0.027	0.838	1.193	0.79
How often do you eat these foods? [Lemon]	-.212c	-2.395	0.019	-0.235	0.892	1.12	0.845
How often do you eat these foods? [Potato]	-.231c	-2.744	0.007	-0.267	0.968	1.033	0.91
How often do you eat these foods? [Rice]	-.174c	-2.046	0.043	-0.202	0.979	1.022	0.908
How often do you eat these foods? [Oats]	-.131c	-1.532	0.129	-0.153	0.982	1.018	0.912
How often do you eat these foods? [Barley]	-.068c	-0.772	0.442	-0.078	0.938	1.066	0.867
How often do you eat these foods? [Wheat]	-.023c	-0.258	0.797	-0.026	0.965	1.037	0.891
How often do you eat these foods? [Corn]	-.098c	-1.132	0.26	-0.114	0.971	1.03	0.905
How often do you eat these foods? [Millet]	.023c	0.248	0.804	0.025	0.832	1.202	0.784
How often do you eat these foods? [Beans]	-.042c	-0.472	0.638	-0.048	0.933	1.072	0.897
How often do you eat these foods? [Lentils]	-.082c	-0.928	0.356	-0.093	0.943	1.061	0.898
How often do you eat these foods? [Cocoa]	-.042c	-0.477	0.634	-0.048	0.935	1.07	0.864
How often do you eat these foods? [Almonds]	.036c	0.393	0.695	0.04	0.874	1.144	0.808
How often do you eat these foods? [Walnuts]	-.014c	-0.155	0.877	-0.016	0.909	1.1	0.845
How often do you eat these foods? [Peanuts]	-.067c	-0.753	0.453	-0.076	0.919	1.088	0.853
How often do you eat these foods? [Chia seeds]	-.116c	-1.233	0.22	-0.124	0.821	1.218	0.764
How often do you eat these foods? [Pine nuts]	-.013c	-0.143	0.887	-0.014	0.853	1.173	0.799
How often do you eat these foods? [Avocado]	-.189c	-2.231	0.028	-0.22	0.979	1.022	0.91
How often do you eat these foods? [Green apple]	-.090c	-1.046	0.298	-0.105	0.98	1.02	0.908
How often do you eat these foods? [Artichoke]	.045c	0.496	0.621	0.05	0.887	1.128	0.826
How often do you eat these foods? [Asparagus]	-.027c	-0.304	0.762	-0.031	0.915	1.093	0.857

How often do you eat these foods? [Corn]	-.012c	-0.133	0.895	-0.013	0.933	1.072	0.895
How often do you eat these foods? [Honey]	-.039c	-0.433	0.666	-0.044	0.888	1.126	0.873
How often do you eat these foods? [Sweet potato]	.002c	0.025	0.98	0.003	0.95	1.052	0.889
How often do you eat these foods? [Cabbage]	-.037c	-0.436	0.664	-0.044	0.994	1.006	0.918
How often do you eat these foods? [Cucumber]	-.086c	-1.01	0.315	-0.102	0.999	1.001	0.923
How often do you eat these foods? [Green plantain]	-.190c	-2.203	0.03	-0.217	0.942	1.061	0.879
How often do you eat these foods? [Banana]	-.220c	-2.634	0.01	-0.257	0.984	1.016	0.91
How often do you eat these foods? [Passion fruit]	-.170c	-1.931	0.056	-0.191	0.917	1.09	0.883
How often do you eat these foods? [Cassava]	-.133c	-1.54	0.127	-0.154	0.963	1.038	0.91
How often do you eat these foods? [Green mango]	-.175c	-2.034	0.045	-0.201	0.95	1.052	0.897
How often do you eat these foods? [Ripe mango]	-.062c	-0.717	0.475	-0.072	0.973	1.028	0.908
How often do you eat these foods? [Caimito]	.019c	0.215	0.83	0.022	0.916	1.092	0.863
How often do you eat these foods? [Carambola]	-.051c	-0.556	0.58	-0.056	0.858	1.166	0.812
How often do you consume these spices? [Parsley]	.099c	1.09	0.279	0.109	0.884	1.132	0.851
How often do you consume these spices? [Cilantro]	.009c	0.108	0.914	0.011	0.998	1.002	0.922
How often do you consume these spices? [Oregano]	.025c	0.28	0.78	0.028	0.947	1.056	0.883
How often do you consume these spices? [Garlic]	-.163c	-1.929	0.057	-0.191	0.996	1.004	0.92
How often do you consume these spices? [Cloves]	.115c	1.309	0.194	0.131	0.933	1.072	0.874
How often do you consume these spices? [Paprika]	.039c	0.433	0.666	0.044	0.924	1.083	0.859
How often do you consume these spices? [Marjoram]	.053c	0.577	0.566	0.058	0.882	1.134	0.816
How often do you consume these spices? [Achiote]	.093c	1.055	0.294	0.106	0.945	1.058	0.875
How often do you consume these spices? [Ginger]	.093c	1.013	0.314	0.102	0.858	1.165	0.797
How often do you consume these spices? [Saffron]	.002c	0.026	0.979	0.003	0.871	1.148	0.809
How often do you consume these spices? [Anise]	.109c	1.236	0.219	0.124	0.927	1.079	0.865
How often do you consume these spices? [Bay leaves]	.078c	0.858	0.393	0.086	0.881	1.135	0.815
How often do you consume these spices? [Thyme]	.119c	1.286	0.202	0.129	0.849	1.178	0.787
How often do you consume these spices? [Rosemary]	.078c	0.83	0.408	0.084	0.836	1.196	0.78
How often do you consume these spices? [Turmeric]	.196c	2.075	0.041	0.205	0.794	1.259	0.74
How often do you consume these spices? [Basil]	.136c	1.448	0.151	0.145	0.818	1.222	0.759
How often do you consume these spices? [Cumin]	.002c	0.021	0.984	0.002	0.942	1.062	0.894
How often do you consume these spices? [Cinnamon]	.021c	0.24	0.811	0.024	0.94	1.063	0.894
How often do you consume these spices? [Sesame]	.192c	2.203	0.03	0.217	0.924	1.082	0.882
How often do you consume these spices? [Salt]	-.038c	-0.442	0.659	-0.045	0.979	1.022	0.904
How often do you consume these spices? [Vanilla]	.070c	0.752	0.454	0.076	0.841	1.189	0.841
How often do you consume these spices? [Crumbled Magui (chicken bouillon cubes)]	-.034c	-0.388	0.699	-0.039	0.934	1.07	0.874

How often do you consume these spices? [Regular bouillon cubes]	-.073c	-0.835	0.406	-0.084	0.965	1.037	0.905
How often do you consume these spices? [Tricompleto (garlic, cumin, pepper)]	.094c	1.086	0.28	0.109	0.978	1.023	0.914
How often do you consume fermented foods? [Pickled olives]	.043c	0.444	0.658	0.045	0.775	1.29	0.749
How often do you consume fermented foods? [Blue cheese]	-.021c	-0.211	0.834	-0.021	0.735	1.361	0.735
How often do you consume fermented foods? [Parmesan cheese]	-.026c	-0.284	0.777	-0.029	0.859	1.165	0.859
How often do you consume fermented foods? [Guarapo]	-.013c	-0.15	0.881	-0.015	0.914	1.094	0.889
How often do you consume fermented foods? [Chicha]	-.029c	-0.319	0.751	-0.032	0.89	1.123	0.873
How often do you consume fermented foods? [ChampÃºs]	-.001c	-0.016	0.988	-0.002	0.853	1.172	0.803
How often do you consume fermented foods? [Cream cheese]	-.036c	-0.398	0.691	-0.04	0.884	1.131	0.874
How often do you consume fermented foods? [Quesillo oaxaca]	.154c	1.386	0.169	0.139	0.583	1.716	0.58
How often do you consume fermented foods? [Queso costeño]	-.137c	-1.575	0.118	-0.157	0.948	1.055	0.898
How often do you consume fermented foods? [Peasant cheese (kneaded)]	-.060c	-0.68	0.498	-0.069	0.947	1.055	0.884
How often do you consume fermented foods? [Coastal butter cheese]	-.118c	-1.326	0.188	-0.133	0.912	1.097	0.884
How often do you consume fermented foods? [Coastal whey]	-.019c	-0.212	0.832	-0.021	0.919	1.088	0.894
How often do you consume fermented foods? [Rikur yogurt]	.018c	0.188	0.851	0.019	0.849	1.177	0.806
How often do you consume fermented foods? [Natilla]	-.018c	-0.205	0.838	-0.021	0.912	1.096	0.874
How often do you consume? [Orange juice]	-.043c	-0.5	0.618	-0.05	0.996	1.004	0.922
How often do you consume? [Mango juice]	-.008c	-0.084	0.933	-0.008	0.891	1.122	0.861
How often do you consume? [Apple juice]	-.070c	-0.778	0.438	-0.078	0.905	1.105	0.836
How often do you consume? [Cherry juice]	-.050c	-0.543	0.589	-0.055	0.88	1.137	0.82
How often do you consume? [Activia Yogurt]	.168c	1.799	0.075	0.179	0.82	1.219	0.82
How often do you consume? [Svelty Actifibras Powdered Milk, Nestlé]	.155c	1.606	0.112	0.16	0.772	1.296	0.772
How often do you consume? [Yogo yogo]	-.016c	-0.167	0.868	-0.017	0.84	1.19	0.84
How often do you consume? [Alpina]	.009c	0.1	0.921	0.01	0.833	1.2	0.833
How often do you consume? [Corozo]	.009c	0.099	0.921	0.01	0.954	1.048	0.893
How often do you consume? [Sinuberase]	.094c	0.293	0.77	0.03	0.072	13.895	0.071
How often do you consume? [Probiotix]	.269c	1.059	0.292	0.106	0.113	8.86	0.111
How often do you consume? [Enterogermina]	-.015c	-0.126	0.9	-0.013	0.517	1.933	0.507
How often do you consume? [Alflorex]	.244c	0.959	0.34	0.096	0.113	8.855	0.111

How often do you consume it? [Bioflor]	.177c	0.872	0.385	0.088	0.178	5.614	0.178
How often do you consume it? [Inmunofort]	.446c	1.407	0.163	0.141	0.072	13.892	0.072
How often do you consume it? [Biogaia]	.068c	0.259	0.796	0.026	0.108	9.24	0.108
How often do you consume it? [Bifidolac]	.767c	1.847	0.068	0.183	0.041	24.175	0.041
How often do you consume it? [Multiflora]	.010c	0.054	0.957	0.005	0.202	4.949	0.191
How often do you consume it? [Vivera]	.231c	1.446	0.151	0.144	0.283	3.53	0.283
How often do you consume these drinks? [Coffee]	-.060c	-0.685	0.495	-0.069	0.955	1.047	0.899
How often do you consume these beverages? [Green tea]	.154c	1.796	0.076	0.179	0.967	1.034	0.896
How often do you consume these beverages? [Black tea]	.174c	1.974	0.051	0.196	0.914	1.094	0.866
How often do you consume these beverages? [Linden tea]	.206c	2.365	0.02	0.232	0.921	1.086	0.868
How often do you consume these beverages? [Lemon balm tea]	.143c	1.632	0.106	0.163	0.937	1.067	0.89
How often do you consume these beverages? [Chamomile]	.172c	2.016	0.047	0.2	0.974	1.027	0.915
How often do you consume these beverages? [Spearmint tea]	.132c	1.513	0.133	0.151	0.95	1.053	0.878
How often do you consume these beverages? [Cinnamon]	.067c	0.762	0.448	0.077	0.942	1.061	0.895
How often do you consume these beverages? [Honey water]	.096c	1.093	0.277	0.11	0.949	1.054	0.899
How often do you consume these beverages? [Agua miel]	.055c	0.578	0.564	0.058	0.809	1.236	0.779
How often do you consume these beverages? [Aguardiente]	.092c	0.917	0.362	0.092	0.728	1.373	0.721
How often do you consume these beverages? [Medellin]	.074c	0.763	0.448	0.077	0.787	1.27	0.779
How often do you consume these beverages? [8-year-old rum]	.066c	0.672	0.503	0.068	0.77	1.298	0.754
How often do you consume these beverages? [Rum]	.096c	0.943	0.348	0.095	0.698	1.433	0.655
How often do you consume these beverages? [Viche]	.100c	1.131	0.261	0.113	0.932	1.073	0.861
How often do you consume these beverages? [Cinnamon guarapo]	.027c	0.254	0.8	0.026	0.663	1.507	0.65
How often do you eat fast food? [Buñuelos]	.019c	0.173	0.863	0.018	0.59	1.696	0.59
How often do you eat fast food? [Pasteles]	.048c	0.409	0.684	0.041	0.541	1.849	0.541
How often do you eat fast food? [Arepas]	.125c	0.997	0.321	0.1	0.461	2.168	0.447
Sex	.009e	0.116	0.908	0.012	0.985	1.015	0.838
Age	-.006e	-0.068	0.946	-0.007	0.854	1.171	0.783
Weight (kg)	-.036e	-0.457	0.649	-0.047	0.988	1.012	0.845
BMI	-.010e	-0.13	0.897	-0.013	0.982	1.018	0.846
Do you have overweight or obesity?	.149e	1.828	0.071	0.183	0.913	1.095	0.803
Do you have type 2 diabetes mellitus? (High blood sugar)	-.012e	-0.147	0.883	-0.015	0.978	1.023	0.843
Do you have hypertension? (High blood pressure)	-.039e	-0.468	0.641	-0.048	0.921	1.086	0.843
Do you have high cholesterol?	.066e	0.819	0.415	0.083	0.969	1.031	0.835

Do you have dyslipidemia (high triglycerides)?	.047e	0.579	0.564	0.059	0.957	1.045	0.848
How often do you eat these foods? [Grapes]	-.088e	-1.045	0.299	-0.106	0.87	1.15	0.811
How often do you eat these foods? [Plums]	.089e	0.923	0.358	0.094	0.667	1.499	0.667
How often do you eat these foods? [Blueberries]	-.024e	-0.27	0.788	-0.028	0.774	1.292	0.774
How often do you eat these foods? [Peaches]	-.035e	-0.374	0.709	-0.038	0.714	1.4	0.714
How often do you eat these foods? [Raspberries]	.027e	0.298	0.766	0.03	0.775	1.29	0.775
How often do you eat these foods? [Blueberries]	.019e	0.213	0.832	0.022	0.823	1.215	0.739
How often do you eat these foods? [Grapefruit]	.053e	0.589	0.557	0.06	0.78	1.282	0.78
How often do you eat these foods? [Kiwi]	.012e	0.135	0.893	0.014	0.749	1.335	0.749
How often do you eat these foods? [Oranges]	.014e	0.167	0.868	0.017	0.909	1.1	0.824
How often do you eat these foods? [Guava]	.052e	0.603	0.548	0.061	0.841	1.19	0.791
How often do you eat these foods? [Strawberries]	-.031e	-0.348	0.729	-0.035	0.809	1.236	0.798
How often do you eat these foods? [Pomegranates]	-.025e	-0.279	0.781	-0.028	0.757	1.321	0.757
How often do you eat these foods? [Cherry]	.063e	0.706	0.482	0.072	0.791	1.265	0.791
How often do you eat these foods? [Mandarin Oranges]	-.034e	-0.366	0.715	-0.037	0.731	1.369	0.731
How often do you eat these foods? [Yellow Bananas]	.000e	0.003	0.998	0	0.781	1.28	0.775
How often do you eat these foods? [Limes]	.003e	0.027	0.978	0.003	0.747	1.338	0.747
How often do you eat these foods? [Mamey]	.013e	0.142	0.887	0.014	0.745	1.342	0.745
How often do you eat these foods? [Soursop]	-.101e	-1.078	0.284	-0.109	0.702	1.424	0.702
How often do you eat these foods? [Papaya]	-.105e	-1.123	0.264	-0.114	0.705	1.418	0.705
How often do you eat these foods? [Pitahaya]	-.015e	-0.173	0.863	-0.018	0.789	1.268	0.784
How often do you eat these foods? [Mushrooms]	-.051e	-0.609	0.544	-0.062	0.891	1.122	0.773
How often do you eat these foods? [Peppers]	.120e	1.309	0.194	0.132	0.728	1.373	0.728
How often do you eat these foods? [Carrots]	.040e	0.451	0.653	0.046	0.797	1.254	0.759
How often do you eat these foods? [Chili peppers]	.025e	0.305	0.761	0.031	0.915	1.093	0.839
How often do you eat these foods? [Lettuce]	.014e	0.166	0.868	0.017	0.838	1.194	0.816
How often do you eat these foods? [Radish]	-.041e	-0.43	0.668	-0.044	0.671	1.49	0.671
How often do you eat these foods? [Celery]	-.002e	-0.027	0.979	-0.003	0.863	1.159	0.807
How often do you eat these foods? [Green tomato]	.054e	0.548	0.585	0.056	0.65	1.538	0.65
How often do you eat these foods? [Prickly pear]	-.014e	-0.142	0.888	-0.014	0.657	1.523	0.629
How often do you eat these foods? [Sapote]	-.023e	-0.215	0.83	-0.022	0.554	1.805	0.554
How often do you eat these foods? [Red onion]	.164e	1.278	0.204	0.129	0.374	2.673	0.362
How often do you eat these foods? [Spring onion]	-.030e	-0.266	0.791	-0.027	0.492	2.032	0.465
How often do you eat these foods? [Broccoli]	-.030e	-0.304	0.761	-0.031	0.642	1.558	0.642
How often do you eat these foods? [Green beans]	-.037e	-0.365	0.716	-0.037	0.612	1.635	0.612
How often do you eat these foods? [Pumpkin]	.059e	0.618	0.538	0.063	0.696	1.437	0.696

How often do you eat these foods? [Spinach]	.146e	1.458	0.148	0.147	0.613	1.631	0.613
How often do you eat these foods? [Cauliflower]	-.016e	-0.159	0.874	-0.016	0.647	1.546	0.647
How often do you eat these foods? [Lemon]	-.092e	-1.002	0.319	-0.102	0.73	1.37	0.73
How often do you eat these foods? [Potato]	-.094e	-1.012	0.314	-0.103	0.724	1.381	0.693
How often do you eat these foods? [Rice]	-.057e	-0.644	0.521	-0.066	0.795	1.257	0.751
How often do you eat these foods? [Oats]	-.099e	-1.191	0.237	-0.121	0.887	1.128	0.847
How often do you eat these foods? [Barley]	-.023e	-0.262	0.794	-0.027	0.837	1.195	0.789
How often do you eat these foods? [Wheat]	-.074e	-0.874	0.384	-0.089	0.868	1.152	0.799
How often do you eat these foods? [Corn]	-.119e	-1.45	0.15	-0.146	0.91	1.099	0.838
How often do you eat these foods? [Millet]	-.003e	-0.034	0.973	-0.003	0.795	1.257	0.742
How often do you eat these foods? [Beans]	-.042e	-0.47	0.639	-0.048	0.789	1.268	0.789
How often do you eat these foods? [Lentils]	-.109e	-1.252	0.214	-0.127	0.816	1.226	0.802
How often do you eat these foods? [Cocoa]	-.055e	-0.581	0.563	-0.059	0.697	1.435	0.697
How often do you eat these foods? [Almonds]	-.034e	-0.345	0.731	-0.035	0.637	1.571	0.637
How often do you eat these foods? [Walnuts]	-.050e	-0.529	0.598	-0.054	0.688	1.454	0.688
How often do you eat these foods? [Peanuts]	-.107e	-1.145	0.255	-0.116	0.704	1.421	0.704
How often do you eat these foods? [Chia seeds]	-.156e	-1.729	0.087	-0.174	0.746	1.34	0.723
How often do you eat these foods? [Pine nuts]	-.066e	-0.736	0.463	-0.075	0.769	1.3	0.761
How often do you eat these foods? [Avocado]	-.146e	-1.708	0.091	-0.172	0.834	1.198	0.817
How often do you eat these foods? [Green apple]	-.085e	-0.982	0.329	-0.1	0.818	1.222	0.816
How often do you eat these foods? [Artichoke]	.024e	0.278	0.782	0.028	0.809	1.237	0.776
How often do you eat these foods? [Asparagus]	-.071e	-0.801	0.425	-0.081	0.787	1.271	0.781
How often do you eat these foods? [Corn]	-.033e	-0.352	0.725	-0.036	0.712	1.404	0.712
How often do you eat these foods? [Honey]	-.098e	-1.102	0.273	-0.112	0.78	1.281	0.78
How often do you eat these foods? [Sweet potato]	-.026e	-0.278	0.782	-0.028	0.719	1.39	0.719
How often do you eat these foods? [Cabbage]	.006e	0.058	0.954	0.006	0.595	1.68	0.595
How often do you eat these foods? [Cucumber]	.025e	0.209	0.835	0.021	0.439	2.279	0.439
How often do you eat these foods? [Green plantain]	-.105e	-1.189	0.237	-0.12	0.783	1.277	0.776
How often do you eat these foods? [Banana]	-.118e	-1.301	0.197	-0.132	0.752	1.33	0.709
How often do you eat these foods? [Passion fruit]	-.092e	-1.054	0.294	-0.107	0.811	1.234	0.811
How often do you eat these foods? [Cassava]	-.106e	-1.263	0.21	-0.128	0.868	1.152	0.837
How often do you eat these foods? [Green mango]	-.144e	-1.703	0.092	-0.171	0.851	1.175	0.838
How often do you eat these foods? [Ripe mango]	-.015e	-0.184	0.854	-0.019	0.883	1.133	0.83
How often do you eat these foods? [Caimito]	-.046e	-0.506	0.614	-0.052	0.744	1.345	0.734
How often do you eat these foods? [Carambola]	-.070e	-0.753	0.453	-0.077	0.729	1.372	0.729
How often do you eat these spices? [Parsley]	.032e	0.352	0.726	0.036	0.736	1.359	0.736

How often do you consume these spices? [Cilantro]	-.016e	-0.198	0.844	-0.02	0.959	1.043	0.846
How often do you consume these spices? [Oregano]	-.015e	-0.178	0.859	-0.018	0.919	1.088	0.825
How often do you consume these spices? [Garlic]	-.118e	-1.387	0.169	-0.14	0.846	1.182	0.817
How often do you consume these spices? [Cloves]	.042e	0.469	0.64	0.048	0.786	1.272	0.742
How often do you consume these spices? [Paprika]	.024e	0.279	0.781	0.028	0.874	1.144	0.803
How often do you consume these spices? [Marjoram]	-.063e	-0.696	0.488	-0.071	0.765	1.308	0.76
How often do you consume these spices? [Achiote]	.042e	0.426	0.671	0.043	0.652	1.534	0.644
How often do you consume these spices? [Ginger]	.025e	0.254	0.8	0.026	0.641	1.56	0.641
How often do you consume these spices? [Saffron]	-.093e	-1.01	0.315	-0.103	0.723	1.382	0.723
How often do you consume these spices? [Anise]	-.029e	-0.299	0.766	-0.03	0.673	1.486	0.636
How often do you consume these spices? [Bay leaves]	-.016e	-0.165	0.87	-0.017	0.7	1.428	0.7
How often do you consume these spices? [Thyme]	.056e	0.579	0.564	0.059	0.661	1.512	0.661
How often do you consume these spices? [Rosemary]	.055e	0.584	0.56	0.06	0.714	1.401	0.714
How often do you consume these spices? [Turmeric]	.102e	1.078	0.284	0.109	0.694	1.44	0.694
How often do you consume these spices? [Basil]	.061e	0.65	0.517	0.066	0.718	1.392	0.718
How often do you consume these spices? [Cumin]	-.028e	-0.296	0.768	-0.03	0.713	1.402	0.713
How often do you consume these spices? [Cinnamon]	-.115e	-1.183	0.24	-0.12	0.649	1.54	0.617
How often do you consume these spices? [Salt]	-.008e	-0.1	0.921	-0.01	0.971	1.03	0.849
How often do you consume these spices? [Vanilla]	-.042e	-0.437	0.663	-0.045	0.672	1.489	0.672
How often do you consume these spices? [Crumbled Maggi (chicken bouillon cubes)]	-.016e	-0.185	0.854	-0.019	0.792	1.262	0.792
How often do you consume these spices? [Regular Maggi (chicken bouillon cubes)]	-.035e	-0.413	0.68	-0.042	0.862	1.16	0.842
How often do you consume these spices? [Tricompleto (garlic, cumin, pepper)]	.083e	0.995	0.322	0.101	0.879	1.138	0.815
How often do you consume fermented foods? [Pickled olives]	.005e	0.05	0.961	0.005	0.76	1.315	0.715
How often do you consume fermented foods? [Blue cheese]	-.071e	-0.761	0.448	-0.077	0.721	1.388	0.718
How often do you consume fermented foods? [Parmesan cheese]	-.020e	-0.239	0.812	-0.024	0.858	1.166	0.793
How often do you consume fermented foods? [Guarapo]	-.059e	-0.673	0.502	-0.069	0.815	1.226	0.797
How often do you consume fermented foods? [Chicha]	-.096e	-1.028	0.307	-0.104	0.716	1.396	0.716
How often do you consume fermented foods? [ChampÃºs]	-.039e	-0.456	0.649	-0.047	0.837	1.195	0.76
How often do you consume fermented foods? [Cream cheese]	-.073e	-0.845	0.4	-0.086	0.834	1.199	0.83
How often do you consume fermented foods? [Quesillo oaxaca]	.099e	0.949	0.345	0.096	0.573	1.746	0.563

How often do you consume fermented foods? [Queso costeño]	-.039e	-0.458	0.648	-0.047	0.844	1.186	0.822
How often do you consume fermented foods? [Queso campesino (kneaded)]	-.017e	-0.207	0.837	-0.021	0.879	1.138	0.807
How often do you consume fermented foods? [Coastal butter cheese]	-.092e	-1.078	0.284	-0.109	0.853	1.172	0.81
How often do you consume fermented foods? [Coastal whey]	-.004e	-0.048	0.962	-0.005	0.883	1.133	0.823
How often do you consume fermented foods? [Rikur yogurt]	.003e	0.037	0.97	0.004	0.769	1.301	0.756
How often do you consume fermented foods? [Natilla]	-.059e	-0.657	0.513	-0.067	0.777	1.286	0.777
How often do you consume? [Orange juice]	-.053e	-0.664	0.509	-0.068	0.988	1.012	0.848
How often do you consume? [Mango juice]	-.041e	-0.482	0.631	-0.049	0.864	1.157	0.84
How often do you consume? [Apple juice]	-.090e	-1.068	0.288	-0.108	0.868	1.152	0.784
How often do you consume? [Cherry juice]	-.087e	-0.975	0.332	-0.099	0.776	1.289	0.772
How often do you consume? [Activia Yogurt]	.077e	0.827	0.41	0.084	0.723	1.383	0.723
How often do you consume it? [Svelty Actifibras powdered milk, Nestlé]	.104e	1.156	0.251	0.117	0.758	1.32	0.758
How often do you consume it? [Yogo yogo]	-.094e	-1.041	0.301	-0.106	0.763	1.311	0.763
How often do you consume it? [Alpina]	-.029e	-0.323	0.747	-0.033	0.795	1.258	0.795
How often do you consume it? [Corozo]	-.048e	-0.555	0.58	-0.057	0.852	1.174	0.791
How often do you consume it? [Sinuberase]	-.051e	-0.172	0.864	-0.018	0.071	14.123	0.071
How often do you consume it? [Probiotix]	.104e	0.435	0.664	0.044	0.109	9.144	0.109
How often do you consume it? [Enterogermina]	-.063e	-0.558	0.578	-0.057	0.484	2.065	0.484
How often do you consume it? [Alflorex]	.098e	0.413	0.68	0.042	0.11	9.053	0.11
How often do you consume it? [Bioflor]	.113e	0.597	0.552	0.061	0.175	5.718	0.175
How often do you consume it? [Inmunofort]	.428e	1.464	0.147	0.148	0.072	13.954	0.072
How often do you consume it? [Biogaia]	-.011e	-0.044	0.965	-0.004	0.107	9.326	0.107
How often do you consume it? [Bifidolac]	.568e	1.466	0.146	0.148	0.041	24.537	0.041
How often do you consume it? [Multiflora]	-.020e	-0.115	0.909	-0.012	0.201	4.986	0.189
How often do you consume? [Herbal tea]	.118e	0.775	0.44	0.079	0.268	3.736	0.268
How often do you consume these beverages? [Coffee]	-.009e	-0.109	0.913	-0.011	0.921	1.086	0.838
How often do you consume these beverages? [Green tea]	.138e	1.699	0.093	0.171	0.919	1.088	0.833
How often do you consume these beverages? [Black tea]	.114e	1.358	0.178	0.137	0.876	1.141	0.817
How often do you consume these beverages? [Linden tea]	.158e	1.925	0.057	0.193	0.894	1.118	0.816
How often do you consume these beverages? [Lemon balm tea]	.130e	1.56	0.122	0.157	0.874	1.144	0.828
How often do you consume these beverages? [Chamomile]	.134e	1.642	0.104	0.165	0.908	1.102	0.819
How often do you consume these beverages? [Spearmint tea]	.089e	1.077	0.284	0.109	0.897	1.115	0.824

How often do you consume these beverages? [Cinnamon]	-.003e	-0.032	0.974	-0.003	0.826	1.21	0.771
How often do you consume these beverages? [Honey water]	.063e	0.753	0.453	0.077	0.891	1.122	0.828
How often do you consume these beverages? [Agua miel]	.096e	1.094	0.277	0.111	0.801	1.249	0.766
How often do you consume these beverages? [Medellin]	.116e	1.256	0.212	0.127	0.717	1.395	0.717
How often do you consume these beverages? [8-year-old rum]	.104e	1.159	0.249	0.117	0.763	1.311	0.763
How often do you consume these beverages? [Rum]	.095e	1.04	0.301	0.106	0.742	1.348	0.742
How often do you consume these beverages? [Viche]	.088e	0.927	0.356	0.094	0.695	1.438	0.622
How often do you consume these beverages? [Cinnamon guarapo]	.108e	1.182	0.24	0.12	0.739	1.353	0.739
How often do you eat fast food? [Fritanga]	-.017e	-0.172	0.864	-0.018	0.656	1.524	0.641
How often do you eat fast food? [Buñuelos]	.012e	0.114	0.91	0.012	0.587	1.703	0.587
How often do you eat fast food? [Pasteles]	.010e	0.095	0.924	0.01	0.534	1.872	0.534
How often do you eat fast food? [Arepas]	.105e	0.909	0.366	0.092	0.461	2.171	0.442
